# c-Myb regulates matrix metalloproteinases 1/9, and cathepsin D: implications for matrix-dependent breast cancer cell invasion and metastasis

**DOI:** 10.1186/1476-4598-11-15

**Published:** 2012-03-23

**Authors:** Lucia Knopfová, Petr Beneš, Lucie Pekarčíková, Markéta Hermanová, Michal Masařík, Zuzana Pernicová, Karel Souček, Jan Šmarda

**Affiliations:** 1 Department of Experimental Biology, Faculty of Science, Masaryk University, Brno, Czech Republic; 2 International Clinical Research Center, CBCE, St. Anne's University Hospital, Brno, Czech Republic; 3 First Department of Pathological Anatomy, St. Anne's University Hospital and Faculty of Medicine, Masaryk University, Brno, Czech Republic; 4 Department of Pathological Physiology, Faculty of Medicine, Masaryk University, Brno, Czech Republic; 5 Department of Cytokinetics, Academy of Sciences of the Czech Republic, Institute of Biophysics, Brno, Czech Republic

**Keywords:** c-Myb, Metastasis, Breast cancer, Matrix metalloproteinase, Cathepsin D, Extracellular matrix

## Abstract

**Background:**

The c-Myb transcription factor is essential for the maintenance of stem-progenitor cells in bone marrow, colon epithelia, and neurogenic niches. c-Myb malfunction contributes to several types of malignancies including breast cancer. However, the function of c-Myb in the metastatic spread of breast tumors remains unexplored. In this study, we report a novel role of c-Myb in the control of specific proteases that regulate the matrix-dependent invasion of breast cancer cells.

**Results:**

Ectopically expressed c-Myb enhanced migration and ability of human MDA-MB-231 and mouse 4T1 mammary cancer cells to invade Matrigel but not the collagen I matrix *in vitro*. c-Myb strongly increased the expression/activity of cathepsin D and matrix metalloproteinase (MMP) 9 and significantly downregulated MMP1. The gene coding for cathepsin D was suggested as the c-Myb-responsive gene and downstream effector of the migration-promoting function of c-Myb. Finally, we demonstrated that c-Myb delayed the growth of mammary tumors in BALB/c mice and affected the metastatic potential of breast cancer cells in an organ-specific manner.

**Conclusions:**

This study identified c-Myb as a matrix-dependent regulator of invasive behavior of breast cancer cells.

## Background

Metastatic disease is the main cause of death in cancer patients. The interaction of tumor cells with local stroma plays a critical role in metastatic dissemination and determines metastasis organotropism. Identification of the genes providing cancer cells with the abilities to disseminate to specific organs is essential for targeting metastatic cells to improve patient survival.

The c-Myb protein is a transcription factor that plays a key role in regulating the proliferation/differentiation of progenitor cells in bone marrow, colonic crypts, and neurogenic niches [1]. c-*myb *was originally identified as a cellular homolog of v-*myb*, the transforming retroviral oncogene linked to avian leukemia [2]. Since then, c-*myb *has been characterized as an oncogene in several human tumor types [3-7], including breast cancer [8-10]. The role of c-Myb in stimulating cell proliferation, suppressing differentiation, and apoptosis is well established [1,11]. However, only a few reports concerning the role of c-Myb in controlling tumor invasion have been reported. First, genes coding for some of the proinvasive factors, such as CXCL12 and CXCR4, were identified as c-Myb targets [12-14]. Second, c-Myb is involved in the regulation of epithelial-to-mesenchymal transition (EMT) and invasion in neuroblastoma, colon carcinoma, and embryonic kidney cells through the upregulation of the transcription repressor Slug [15]. Third, in hepatocellular carcinoma cells, c-Myb promotes cell invasion via the upregulation of osteopontin [16]. Interestingly, the depletion of endogenous c-*myb *in MCF7 breast cancer cells increased tumorigenesis *in vitro *and *in vivo*, suggesting that c-Myb acts as a tumor suppressor in breast cancer [17]. However, how c-Myb controls breast carcinoma progression and dissemination remains unresolved. To gain insight into this process, we generated variants of mammary carcinoma cell lines overexpressing c-*myb *and investigated their migratory/invasive and metastatic capabilities. We demonstrated that c-Myb regulates the invasive behavior of breast cancer cells in a matrix-dependent manner, possibly via a novel signaling axis causing the deregulation of MMP1, MMP9, and cathepsin D expression.

## Results

### Ectopically expressed c-*myb *increases the migration and invasion of MDA-MB-231 cells through Matrigel *in vitro*

**Figure 1:**
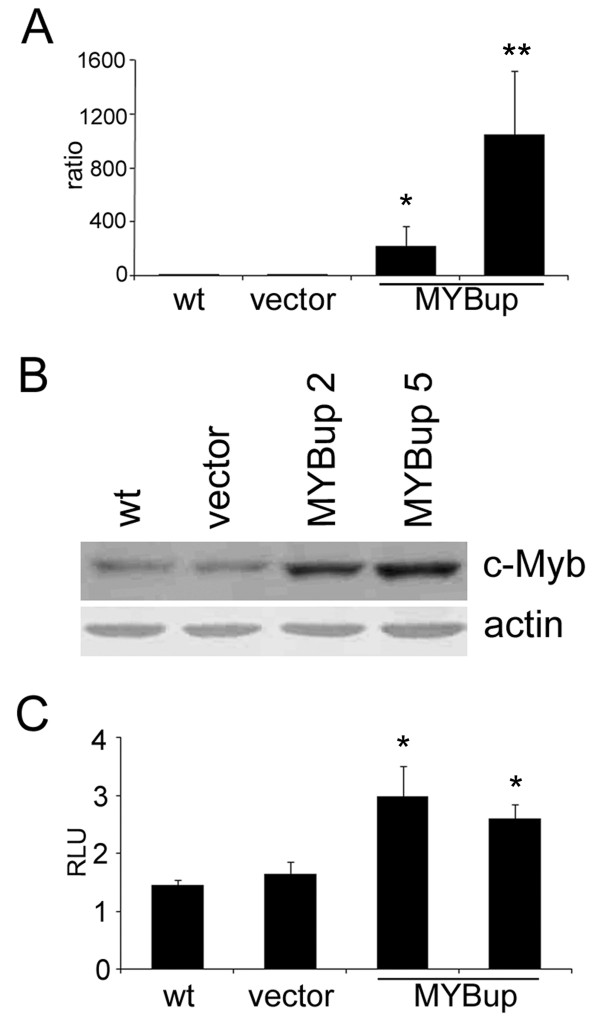
**Derivation of MDA-MB-231 cells overexpressing c-*myb***. The MDA-MB-231 cells were transfected with human c-*myb *cDNA (MYBup) or the *myb*-less vector (vector) by lipofection. Pools of G418-resistant cells were cloned. (**A**) c-*myb *expression was determined in two independent clones by qRT-PCR. *GAPDH *was used as an internal control. (**B**) The c-Myb protein in two independent MYBup clones was determined by immunoblotting, and β-actin was used as a loading control. (**C**) Transactivation by c-Myb was determined by a luciferase assay using p6MBSluc as a reporter. The luciferase activity of each sample was expressed in relative light units and normalized for transfection efficiency according to the β-galactosidase activity. The columns show the average relative luciferase activity from three independent experiments. Error bars indicate standard deviations. Marks * and ** indicate significant (p < 0.05 and p < 0.01, respectively) differences in the relative amounts of c-*myb *mRNA (**A**) and in the luciferase activity (**C**) in the *myb*-less vector-transfected cells and MYBup cells as determined by the *t*-test.

First, variants of MDA-MB-231 cells overexpressing c-*myb *were derived as described in the Material and Methods. The expression of c-*myb *in individual clones of MDA-MB-231MYBup cells significantly exceeded that in non-transfected and *myb*-less vector-transfected controls, as determined by qRT-PCR and immunoblotting (Figure 1A, B). The exogenous c-Myb protein produced in MDA-MB-231MYBup cells was capable of transactivating the model reporter gene from a promoter containing the Myb-binding sites (Figure 1C).

**Figure 2:**
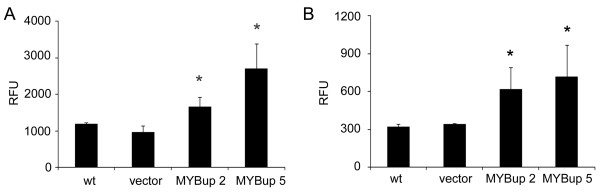
**Overexpression of c-Myb increases the migration and invasiveness of MDA-MB-231 cells through the basement membrane extract/Matrigel**. (**A**) The transmigration assay was performed using Cultrex Cell Invasion Assay. MDA-MB-231MYBup and control cells migrating through a microporous membrane towards the chemoattractant (10% FCS)-containing medium were stained with the Calcein-AM. Cell migration was quantified according to the emitted fluorescence using the Synergy HT microplate reader. The columns show the average values of fluorescence from three independent measurements. (**B**) To determine cell invasiveness, the membrane was covered with a layer of the basement membrane matrix and the experiment was performed as described above. Error bars indicate the standard deviations. Asterisks indicate significant (p < 0.05) differences in the migration (**A**) and invasion (**B**) of the *myb*-less vector-transfected and MYBup cells as determined by the *t*-test.

**Figure 3:**
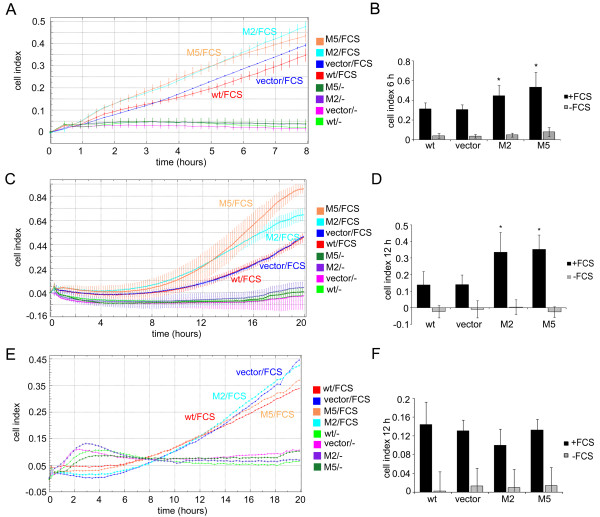
**Kinetics of c-Myb-induced migration and Matrigel invasion of MDA-MB-231MYBup cells**. Cell migration/invasion in real time was analyzed by the xCELLigence RTCA. The control MDA-MB-231 (wt, vector), and the c-*myb*-transfected (M2, M5) cells were seeded at a density of 7.5 × 10^4 ^per well and cultured in the chambers of CIM-plates. For the cell migration experiments, the membranes were left uncoated (**A, B**). For monitoring cell invasion, the membranes were coated with either Matrigel (**C, D**) or collagen I (**E, F**) as described in the Material and Methods. The bottom chambers were filled with either complete medium containing 10% FCS as a chemoattractant or with serum-free medium (-) in control wells. The charts show the representative outcomes of kinetics analysis of cell migration (**A**), Matrigel invasion (**C**) and collagen I invasion (**E**). The panels (**B, D, F**) show the average cell indexes at certain time points (6 h for migration and 12 h for invasion) from seven (migration) and five (invasion) independent measurements. Error bars indicate standard deviations. Asterisks indicate significant (p < 0.05) differences in the migration (**B**) and Matrigel invasion (**D**) of the *myb*-less vector-transfected cells and MYBup cells as determined by the *t*-test.

Next, we assessed the migration and invasion of MDA-MB-231MYBup cells using the Cultrex cell invasion assay (Trevigen, Gaithersberg, MD). We found that exogenous c-Myb enhanced the migration and invasion of MDA-MB-231 cells through Matrigel (Figure 2). To verify these data and to evaluate the kinetics of migration/invasion of the MDA-MB-231MYBup cells in real time, we used the xCELLigence RTCA. The number of migrating or invasive cells can be monitored continuously and expressed as the cell index in this assay [18]. The cell index profiles revealed the rapid onset of c-Myb-induced MDA-MB-231MYBup cell migration (Figure 3A). For statistical analysis, we used the cell index values obtained 6 h after the starting point. The MDA-MB-231MYBup clones were significantly (p < 0.05) more active (1.5- and 1.7-fold) in migration than control cells at this time point (Figure 3B). Similar results were obtained also for 4- and 8-h time points (Additional file 1). Next, the kinetics of cell invasion was analyzed using Matrigel as a surrogate basement membrane (BM) by the same approach. We found that c-Myb substantially accelerated the invasion of MDA-MB-231 cells (Figure 3C). For statistical analysis of invasion rate, the cell index values determined at the 12-h time points were used. Our results demonstrated that c-*myb *overexpression significantly increased (2.4- and 2.5-fold, p < 0.05) the ability of MDA-MB-231 cells to invade Matrigel *in vitro *(Figure 3D). Similar results were obtained also for 10- and 16-h time points (Additional file 1).

To examine whether it is the c-Myb protein that is responsible for increased migration and Matrigel invasiveness of MDA-MB-231MYBup cells, we specifically silenced c-*myb *expression in these cells using siRNA and monitored cell migration/invasion again. We found that cell migration and Matrigel penetration were significantly restrained in the MDA-MB-231MYBup cells transfected with the c-*myb*-specific siRNA (Additional file 2). This confirms that c-Myb upregulates migration and invasiveness of these cells indeed.

### c-Myb does not stimulate cell invasion through the collagen I matrix

To evaluate the invasive capacity of cancer cells within the stromal matrix, the collagen I gel is a suitable matrix barrier [19,20]. Therefore, membranes of the CIM-plates 16 were coated with collagen I matrix, and cell invasion was measured in real time using the xCELLigence RTCA. The cell indexes were determined at the 12-h time points to compare the invasion rates of c-*myb*-overexpressing and control cells. We found that c-Myb did not enhance the ability of MDA-MB-231MYBup cells to penetrate the collagen I matrix barrier (Figure 3E, F). Similar results were obtained also for 10- and 16-h time points (data not shown). This suggests that c-Myb-induced tumor cell invasion *in vitro *depends on composition of the substrate/matrix.

### c-Myb upregulates cathepsin D and MMP9 and downregulates MMP1

**Figure 4:**
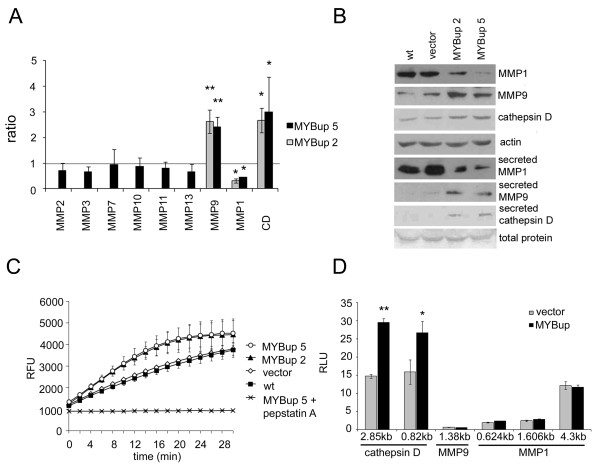
**c-Myb upregulates cathepsin D and MMP9 and downregulates MMP1 in MDA-MB-231MYBup cells**. (**A**) The relative amounts of *MMP2, 3, 7, 10, 11*, and *13 *mRNA in the MYBup 5 and *myb*-less vector-transfected cells were determined by qRT-PCR. The relative amounts of *MMP1, 9 *and cathepsin D mRNAs were also determined in the MYBup 2 clone. Marks * and ** indicate significant (p < 0.05 and p < 0.01, respectively) differences in the relative amounts of *cathepsin D*, *MMP9*, and *MMP1 *mRNA in the *myb*-less vector-transfected and MYBup cells as determined by the *t*-test. The columns show the average values of relative normalized expression of the indicated genes from at least three independent experiments. (**B**) The control (wt, vector) and the MYBup cell protein extracts were analyzed by immunoblotting with anti-MMP1, anti-MMP9, and anti-cathepsin D antibodies. The secretion of cathepsin D, MMP9, and MMP1 was determined in the cell-conditioned medium. Equal amounts of total proteins were loaded. (**C**) To determine the activity of cathepsin D, protein extracts were prepared from harvested cells as described in the Material and Methods. The extent of hydrolysis of the fluorimetric substrate was measured in real time using a Synergy HT microplate reader. To demonstrate the assay specificity, pepstatin A was added to inhibit cathepsin D activity (data shown for MYBup 5 cells). (**D**) Transactivation assays were performed using six variants of luciferase reporters: two for cathepsin D (either containing the 2.85-kb or 0.82-kb promoter sequence), one for MMP9 (1.38 kb), and three for MMP1 (0.624, 1.606, and 4.3 kb). The luciferase activity of each sample was expressed in relative light units and normalized for transfection efficiency according to the β-galactosidase activity. The columns show the average values of relative luciferase activity from three independent measurements. Marks * and ** indicate significant (p < 0.05 and p < 0.01, respectively) differences in the luciferase activities in control cells and MYBup cells as determined by the *t*-test.

Tumor cells acquire ability to surmount extracellular matrix (ECM) barriers by expressing a range of proteases, such as MMPs or cathepsins [21]. Various enzymes are vital for the degradation of either BMs or stromal matrices [22-24]. We found that Matrigel invasion by MDA-MB-231MYBup cells is sensitive to the broad spectrum MMP inhibitor GM6001 (Ilomastat; Additional file 3) suggesting that some of MMPs are responsible for this effect. To further specify the c-Myb-targeted proteases, we first determined the relative amounts of *MMP1*, *2*, *3*, *7*, *9*, *10*, *11*, *13*, and *cathepsin D *mRNAs in MDA-MB-231MYBup cells by qRT-PCR. Out of eight *MMPs *tested, only *MMP9 *(92-kDa gelatinase) was significantly upregulated (2-fold, p < 0.01) in MYBup cells (Figure 4A). Similarly, the *cathepsin D *mRNA was upregulated more than 2-fold (p < 0.05) in the same cells (Figure 4A). Interestingly, the level of *MMP1 *(interstitial collagenase) mRNA dropped by 60% in the MYBup clones (p < 0.05; Figure 4A). The c-Myb-dependent changes of relative amounts of MMP1 and MMP9 mRNAs were reflected also in amounts of MMP1 and MMP9 proteins produced by these cells as determined by immunoblotting (Figure 4B). Whereas c-Myb decreased the expression/secretion of MMP1 in MDA-MB-231MYBup cells, it increased the expression/secretion of MMP9 (Figure 4B). Cathepsin D is another enzyme involved in cell invasion and metastasis, particularly in breast cancer [25]. Several components of ECM, such as fibronectin, proteoglycans, and collagens, are substrates for this lysosomal aspartic protease [25-27]. The level of cathepsin D in MDA-MB-231MYBup cell lysates and conditioned media was determined by immunoblotting. We found that the production of both intracellular and extracellular cathepsin D by MDA-MB-231MYBup cells was higher than that by control cells (Figure 4B). In addition, the enzymatic activity of cathepsin D was also significantly upregulated in the MYBup cells of both clones as determined by the Cathepsin D Assay (Figure 4C). These results document that c-Myb can substantially affect the spectrum and activities of proteases produced by MDA-MB-231 cells.

### c-Myb induces the expression of cathepsin D through a transcriptional mechanism

To elucidate the mechanism by which c-Myb regulates MMP9, MMP1, and cathepsin D in MYBup cells, we first examined whether c-Myb can activate transcription of *cathepsin D*. We performed transactivation assays using two variants of the luciferase reporter: one containing 2.85-kb promoter of *cathepsin D *and the other equipped with the 0.82-kb promoter of *cathepsin D*. The MDA-MB-231MYBup and control cells were transiently transfected with either of the reporter plasmids and harvested 24 h later. Luciferase assays using both reporters revealed significant upregulation of the luciferase expression from the *cathepsin D *promoter in MDA-MB-231MYBup cells (Figure 4D). In contrast, c-Myb failed to transactivate the luciferase reporter from the MMP9 (1.38 kb) and MMP1 (4.3, 1.606, and 0.624 kb) promoter sequences (Figure 4D). This suggests that c-Myb can transactivate the *cathepsin D *promoter but not *MMP9 *and *MMP1 *promoters in transiently transfected plasmid reporters.

### Upregulation of cathepsin D by c-Myb contributes to cell migration and invasion

**Figure 5:**
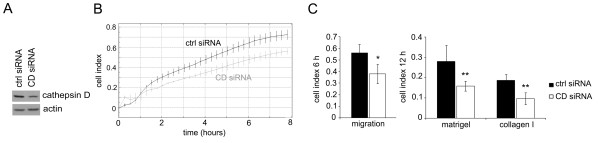
**siRNA-mediated *cathepsin *D silencing reduces the migration and invasion of MDA-MB-231MYBup cells**. MDA-MB-231MYBup cells were transfected with cathepsin D (CD) or control (ctrl) siRNA as described in the Material and Methods. (**A**) The level of cathepsin D protein in these cells was determined by immunoblotting. A representative western blot is presented. (**B**) Migration and invasion activities of the same cells were determined by the xCELLigence RTCA. The chart shows the representative outcomes of the kinetics analysis of cell migration. (**C**) The average cell indexes at the 6-h (migration, left) and 12-h (invasion, right) time points, respectively, from five independent measurements are shown. Error bars indicate standard deviations. Marks * and ** indicate significant (p < 0.05 and p < 0.01, respectively) differences in the migration/invasion of the cells transfected with cathepsin D siRNA and control siRNA as determined by the *t*-test.

To confirm the role of cathepsin D in c-Myb-induced cell migration/invasion, we performed RNAi-mediated silencing of cathepsin D in MDA-MB-231MYBup cells, resulting in effective reduction of the endogenous cathepsin D protein (Figure 5A). The MDA-MB-231MYBup cells with reduced cathepsin D production were substantially less active in migration and invasion through Matrigel and collagen I than MDA-MB-231MYBup cells transfected with control siRNA as determined using the xCELLigence RTCA system (Figure 5B, C). These results document that cathepsin D contributes to the c-Myb-induced migration and invasion of MDA-MB-231 cells.

### c-Myb delays mammary tumor growth and prevents pulmonary metastasis

**Figure 6:**
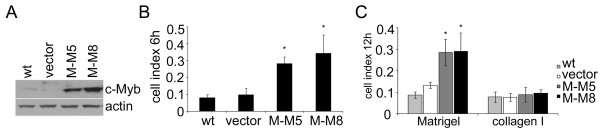
**c-Myb affects cell migration/invasion in murine 4T1 cells similarly as observed in MDA-MB-231 cells**. 4T1 cells were transfected with murine c-*myb *cDNA (M-M5, M-M8) or with the *myb*-less vector (vector) by lipofection. G418-resistant cells were selected and cloned. (**A**) c-*myb *expression in both cell variants (vector and MYBup, respectively) and nontransfected cells (wt) was determined by immunoblotting. The 4T1MYBup variant was tested for migration (**B**) and invasion (**C**) activity using the xCELLigence RTCA as described in Figure 3. The panels represent the average cell indexes at certain time points (6 h for migration and 12 h for invasion) from three (collagen I) and four (migration and Matrigel invasion) independent measurements. Asterisks indicate significant (p < 0.05) differences in the migration (**B**) and Matrigel invasion (**C**) of the *myb*-less vector-transfected cells and MYBup cells as determined by the *t*-test. Error bars indicate standard deviations.

To verify that the effects of c-Myb described in MDA-MB-231 cells are not limited to this particular cell line, we generated variants of murine 4T1 breast cancer cells overexpressing murine c-*myb*. The 4T1MYBup cells were tested for c-*myb *expression, cell migration, and Matrigel/collagen I invasion. Exogenous murine c-Myb was clearly present in 4T1MYBup cells (Figure 6A) and was proficient to activate transcription of the reporter gene from its responsive elements (data not shown) as well as stimulate cell migration and Matrigel invasion (Figure 6B, C). As observed in the MDA-MB-231MYBup cells, the c-Myb-induced invasion of 4T1 cells was limited to Matrigel matrices, and it did not occur in the collagen I matrices (Figure 6C).

**Figure 7:**
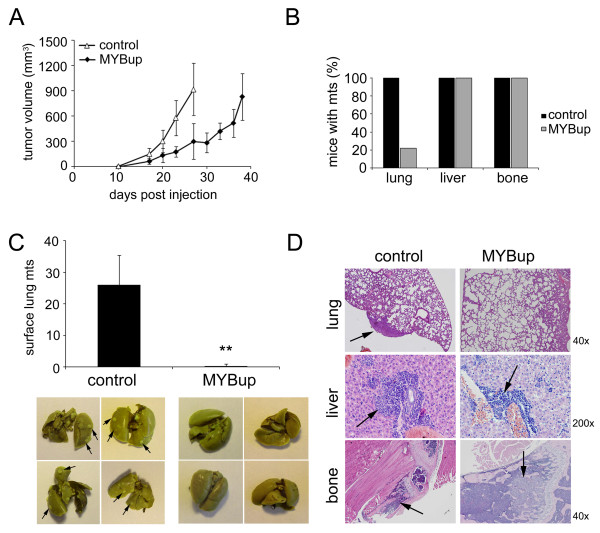
**c-Myb delays mammary tumor growth and prevents the formation of pulmonary metastases**. The tumor growth and spontaneous metastatic ability of the *myb*-less vector-transfected (control) 4T1 and 4T1M-M5 cell variant (MYBup) were determined following orthotopic inoculation into the mammary fat pads of BALB/c mice. (**A**) Tumor growth was monitored twice a week by measuring the tumor length (l) and width (w). Tumor volume was calculated using the equation l × w^2^/2. The mice injected with the MYBup 4T1 variants (n = 9) and control 4T1 cells (n = 9) were euthanized when the mean tumor diameter was approximately 1.2 cm. (**B**) Lungs were removed, rinsed in water, and fixed in Bouin's solution. Liver and bones were harvested and fixed in 10% buffered formalin. The number of lungs with surface metastases was determined using dissecting microscopy, and metastases in liver and bones were determined by histological examination of H&E-stained sections. Percentage of the mice with metastases (mts) developed in lung, liver and bones, respectively, is shown. (**C**) The surface metastatic nodules per lungs (mts) exemplified under the graph were enumerated by examination using dissecting microscopy. Asterisks indicate significant (p < 0.01) differences in the number of lung metastases in the mice injected with MYBup 4T1 variants and control 4T1 cells as determined by the *t*-test. Error bars indicate standard deviations. Representative samples of Bouin's solution-fixed tissues are shown. (**D**) Tissues were processed for paraffin embedding, sectioned, and stained with H&E. Bones were decalcified overnight before embedding. The lung, liver, and bone metastases were identified by light microscopy (arrows).

Next, we wished to investigate behavior of the c-*myb*-overexpressing cancer cells *in vivo*. We used the 4T1MYBup cells in a syngeneic mouse mammary tumor model. The tumor growth and spontaneous metastatic ability of the *myb*-less vector-transfected 4T1 and 4T1MYBup cell variants were compared following orthotopic inoculation into BALB/c mice. The c-*myb*-overexpressing tumors grew at a significantly slower rate than control 4T1 tumors. After 27 days, the average tumor size was larger in control mice injected with cells containing the *myb*-less vector (954 ± 310 mm^3^) than in mice injected with cells expressing c-*myb *(360 ± 212 mm^3^; p < 0.05; Figure 7A). When the mean tumor diameter reached 1.2 cm, the mice were sacrificed, and the tumors were excised. The average tumor weight at harvest was approximately the same in both groups (data not shown). Although all mice in control group developed lung metastases (n = 9), pulmonary metastases were observed only in 22% of mice in the group injected with cells overexpressing c-*myb *(Figure 7B). In addition, the average number of metastatic nodules in lung was significantly (p < 0.01) higher in the control group than in the group injected with the MYBup variants (Figure 7C). Both groups consistently developed liver and bone metastases. The size and number of metastatic lesions in liver and bones were similar in the control and MYBup groups as determined by histological examination (Figure 7B, D; Additional file 4). Taken together, the results implicate that c-Myb participates in the control of breast tumor growth and spontaneous metastasis. Whereas c-Myb delays the outgrowth of primary tumors and reduces their metastatic spread to lungs, it does not affect the frequency or severity of bone and liver metastases.

## Discussion

In this report, we identified the regulation of MMP1/9 and cathepsin D by c-Myb as a novel mechanism of the matrix-specific breast cancer cell invasion. c-Myb has been recently identified as a regulator of tumor cell motility and invasion, and the Slug transcription factor was described as the mediator of the c-Myb-induced mesenchymal-like phenotype in neuroblastoma, colon carcinoma, and embryonic kidney cells [15]. The TGFβ-induced EMT and invasion of estrogen receptor-positive breast cancer cells was also found to be dependent on c-Myb expression [28]. In our study, we confirmed the regulatory role of c-Myb in control of migration and invasion of breast carcinoma cells as well. We also observed upregulation of Slug in the MYBup variants of MDA-MB-231 cells, but we did not detect any c-Myb-dependent changes in expression of either epithelial or mesenchymal cell markers, such as E-cadherin, N-cadherin, or vimentin (Additional file 5). This may presumably result from the mesenchymal-like phenotype of the parental MDA-MB-231 cells. We demonstrated that c-Myb activates transcription of *cathepsin D *in a Slug-independent manner (Additional file 6). This implies that EMT is not an exclusive mode of c-Myb control over cell migration/invasion. In hepatocellular tumor cells, c-Myb stimulated migration/invasion via osteopontin secretion [16]. Activation of the osteopontin promoter by c-Myb was also reported in melanomas [29]. However, expression of the osteopontin gene was not deregulated in the c-*myb*-overexpressing MDA-MB-231 cells (data not shown) implying that c-Myb uses another mechanism to control migration/invasion of breast cancer cells.

There are two main types of ECM in vertebrates: BM and the stromal/interstitial matrix. Components of the BMs include collagen IV, laminin, perlecan, and nidogen. Stromal/interstitial matrices that form the majority of the body connective tissues are composed primarily of fibrillar collagen I. As both BM and stromal matrices represent steric barriers to cell migration, matrix remodeling is a critical prerequisite for metastasis formation. BM extract/Matrigel is frequently used to assess cell invasive capacity *in vitro*. Matrigel invasion *in vitro *is considered to simulate the penetration of BMs underlying epithelial cells or blood vessels by tumor cells *in vivo*. We documented that c-Myb induces the invasion of MDA-MB-231 cells through Matrigel. Conversely, cancer cell invasion through stromal/interstitial matrices composed primarily of fibrillar collagen I was found to be critical for metastasis in several tumor types [22,23]. We found that c-Myb does not activate cell invasion through the collagen I barrier. Modulation of cell invasion by ECM components was described previously [30-32]. Different invasion modes reflecting the specificity of substrates may be attributed to the different requirements on proteolytic systems [22,33]. Therefore, we analyzed the expression of candidate proteases in c-*myb*-overexpressing and control MDA-MB-231 cells and confirmed the differential expression of *MMP1*, *MMP9*, and *cathepsin D*. We confirmed the recent observation by Bhattarai that the c-Myb upregulates MMP9 in breast cancer cells [34]. Our demonstration that c-Myb modulates MMP1 has not been described yet. While cathepsin D and MMP9 were upregulated, the expression of MMP1 was considerably reduced in the MYBup variants. c-Myb-induced Matrigel invasion is sensitive to the broad spectrum MMP inhibitor GM6001 (Ilomastat; Additional file 3). As MMP9 is the only MMP found to be upregulated in MYBup cells and collagen IV, the major component of the BM/Matrigel, is the main substrate for MMP9, we hypothesize that apart from cathepsin D, the MMP9 is an effector of the c-Myb-induced Matrigel invasion. MYBup cells exhibiting high MMP9 activity could transverse the Matrigel matrices more efficiently than controls, even in the absence of MMP1. MMP1 (interstitial collagenase) predominantly cleaves collagen I; therefore, the lack of MMP1 in MDA-MB-231MYBup cells might compromise their penetration through the collagen I matrix. Cathepsin D might secure partial invasion of these cells to collagen I substrates [35], as shown by reduced collagen I penetration of MDA-MB-231MYBup cells with silenced cathepsin D expression (Figure 5).

We propose cathepsin D as a novel downstream mediator of the c-Myb migration/invasion-promoting function. Cathepsin D was suggested as one of the c-Myb-target genes in MCF7 breast cancer cells previously [36]. Our results confirmed this observation in MDA-MB-231 cells. We identified multiple putative Myb-binding sites in the human *cathepsin D *gene promoter using TESS software (Additional file 7). There are conflicting results regarding participation of the cathepsin D in the control of breast cancer cell invasion and metastasis [37-44]. Johnson et al. observed that breast cancer cell invasion did not reflect various release rates of cathepsin D from different subclones of MCF7 cells [41]. Similarly, Glondu et al. modulated cathepsin D expression using antisense inhibition without any effect on invasiveness of breast cancer cells *in vitro *[42]. In contrast, suppression of cathepsin D by antisense oligonucleotides and shRNA in MCF7 and MDA-MB-231 cells, respectively, associated with reduction of their invasion through Matrigel was observed by others [43,44]. The effects of siRNA-mediated silencing of cathepsin D in MDA-MB-231MYBup cells described in our study provide further support to the studies documenting the regulatory function of cathepsin D in breast cancer cell migration and invasiveness. Several hypotheses have been raised concerning the mechanism how cathepsin D exerts its effects in tumors. They include facilitated release of growth factors, degradation of the extracellular matrix to permit invasion of the tumor cells and proteolytic activity-independent stimulation of the tumor cells via protein-binding activity of cathepsin D. We observed that unlike siRNA-mediated suppression of cathepsin D expression, inhibition of its activity using pepstatin A blocked neither migration nor invasion of MDA-MB-231MYBup cells (data not shown). This implies that it is the protein-binding activity of cathepsin D that may be involved in stimulation of the tumor cells. Our results correspond with studies documenting that catalytically inactive mutants of cathepsin D stimulate cell invasion [45,46]. Recently, the LRP1 cell surface receptor was identified as a binding partner for pro-cathepsin D in fibroblasts [47]. Breast cancer cells express LRP1 [48] and the LRP-induced stimulation of cancer cell motility and invasion was described elsewhere [49,50]. Therefore, we can hypothesize that cathepsin D enhances migration/invasion of breast cancer cells via LRP1 signaling.

Despite the upregulation of MMP9 mRNA/protein observed in the cells overexpressing c-*myb*, transient transfection studies demonstrated no transactivation of the reporter gene derived from the MMP9 promoter by c-Myb. Similarly, c-Myb did not repress transcription from the MMP1 promoter, although the level of MMP1 decreased in the MYBup cells. c-Myb was demonstrated to act as a transcriptional transactivator/repressor through specific binding to the promoter regions of target genes in numerous studies. However, there are also reports demonstrating that c-Myb can affect gene expression via indirect mechanisms [51,52]. We studied the stability of the *MMP9/1 *transcripts using actinomycin D and found that c-Myb does not affect the stability of *MMP9/1 *mRNAs (data not shown). We hypothesize that structural and functional differences between transiently transfected plasmid DNA and genomic templates, such as inefficient chromatinization, might explain why c-Myb failed to transactivate/repress the MMP9/1 promoters [53]. There are reports indicating that the Myb-induced chromatin binding and remodeling are essential for the transactivation of its target genes [54,55].

We demonstrated that murine c-Myb regulates migration/invasion of mouse 4T1 cells *in vitro*. 4T1 cells were employed as orthotopic mammary tumor model because they effectively metastasize and display metastatic characteristics similar to those observed in cancer patients [56]. Surprisingly, the c-*myb*-overexpressing 4T1 cells injected into the mammary fat pads of BALB/c mice exhibited delayed tumor growth and no formation of spontaneous pulmonary metastases. Previous studies described that the *myb *genes can function either as oncogenes or as tumor suppressors in different cellular contexts [57] and there are conflicting results concerning the c-*myb *function in breast cancer. Oncogenic role of c-Myb was documented by c-*myb *knockdown in estrogen receptor (ER)-positive breast cancer cell lines resulting in block of estrogen-dependent proliferation [58] and TGFβ-induced invasiveness *in vitro *[28]. Miao et al. have recently published data documenting that established human breast cancer xenografts do not advance when c-Myb is knocked down using shRNA [59]. The tissue-specific deletion of c-*myb *also interferes with mammary tumorigenesis in mouse mammary tumor virus (MMTV)-NEU and MMTV-PyMT mice [59]. On the other hand, there is also report that c-*myb *depletion increases the cell growth and tumorigenesis of MCF7 breast cancer cells both *in vitro *and *in vivo *[17] documenting that c-Myb can also act as tumor suppressor. The data based on human breast cancer microarray expression analysis *in vivo *showed that high c-Myb expression is associated with a good outcome and high differentiation status of the tumors [17]. Similarly, a unique subgroup of estrogen receptor-positive human breast cancers with 100% overall survival, no metastatic potential, and high c-*myb *expression has been described recently [60]. Our study of ectopic c-*myb *overexpression in a mouse orthotopic tumor model supports the view of c-Myb as tumor-suppressor in breast cancer. The c-Myb-controlled expression of Hep27 gene was suggested as a mechanism how c-Myb exerts its tumor-suppressing function in ER-positive breast cancer [17,60]. Hep27 inhibits Mdm2 thereby stabilizes p53 [61]. Therefore, the c-Myb-Hep27-Mdm2-p53 signaling pathway may have functional significance for ER- and p53wt-positive breast cancers cells [61]. Interestingly, good prognosis of patients with ER-negative basal-like subtype of breast tumors with frequently mutated p53 was also associated with high c-*myb *expression [17]. To date, the function of c-Myb in metastasis formation in mouse models has not been clarified. It was demonstrated, however, that c-Myb promotes the bone marrow homing of leukemic cells [15]. Our study showed that overexpressed c-Myb can suppress the formation of pulmonary metastases in a mouse model of mammary carcinoma. We propose that interstitial collagenase may be one of the mediators of the tumor- and metastasis-suppressing function of c-Myb in ER-negative breast cancer cells because interstitial collagenase was clearly downregulated in MDA-MB-231MYBup cells (Figure 4), 4T1MYBup cells (Additional file 8) and in the c-*myb-*overexpressing tumors (Additional file 8). A number of observations support this hypothesis. Upregulation of MMP1 was previously associated with advanced stages of breast cancer, and it was thus suggested as a predictive marker for the development of invasive disease [62]. The shRNA-mediated silencing of MMP1 inhibited growth of MDA-MB-231 cells orthotopically implanted into the mammary fat pads of nude mice [63]. The requirement for MMP1 for breast tumor growth was partly attributed to breakdown of the fibrous stroma within the mammary fat pad and partly to the liberation of growth factors present in ECM [63]. In addition, the MMP1/protease-activated receptor 1 signaling axis promoting mammary tumor growth and metastasis was identified [64-66]. In addition, Gupta et al. reported that MMP1 silencing in combination with *epiregulin (EREG)*, *cyclooxygenase 2 (COX2)*, or *MMP2 *delayed tumor progression [67]. Interestingly, *MMP1*, *EREG*, and *COX2 *are parts of the clinically validated lung metastasis signature [68]. This signature comprises genes marking and mediating breast cancer metastasis to the lungs. *MMP1 *belongs to the family of genes with dual functions conferring both breast tumorigenicity and lung metastagenicity [68,69].

Moreover, MMP1 has been implicated in pulmonary extravasation [67,70]. The barriers to metastasis are distinct in different organs [71,72]. The lung vascular endothelial junctions act as barriers limiting cell passage. In contrast, the bone marrow and liver vasculature consists of capillary vascular channels possessing a discontinuous endothelium [71,73]. Therefore, lung metastases may require robust extravasation such as those provided by *MMP1*, *EREG*, *COX2*, and *MMP2 *[67,71]. In contrast, the requirement for effective extravasation is not principal for bone and liver metastasis [71,73,74]. These findings correspond with our results revealing organ-specific differences in the metastatic ability of c-*myb*-overexpressing cells. The deficit of MMP1 function in the c-*myb*-overexpressing cells may contribute to the selective disadvantage of these cells in lung colonization. However, they can still initiate metastasis in bone and liver.

We demonstrated that enhanced migration and invasion of c-*myb*-overexpressing breast cancer cells through Matrigel *in vitro *does not imply increased metastatic capacity. Inconsistency in proinvasive behavior of tumor cells *in vitro *and metastatic potential in mouse xenograft models *in vivo *was also described previously [75,76]. It was postulated that the capacity of tumor cells to metastasize is determined not only by the inherent characteristics of the cancer cells, such as motility and *in vitro *invasiveness, but it is also modulated by the cancer cell microenvironment, such as ECM deposition, the presence of other proteases, cytokines, growth factors and adaptor proteins [75-77]. Apparently, there are tissue-specific factors in the host that participate in the control of cancer cell invasiveness [78].

## Conclusions

In this report, we identified the regulation of MMP1/9 and cathepsin D by c-Myb as a novel mechanism of breast cancer cell invasion that is dependent on extracellular matrix composition. We documented that c-Myb regulates breast cancer metastases in an organ-specific manner. These findings provide new clues for understanding of the oncogenic/tumor-suppressing functions of c-*myb *and for future clinical applications.

## Methods

### Plasmids

Constructs encoding human (pcDNA3-hcMYB) and mouse c-Myb proteins (pcDNA3-mcMYB) were kindly provided by J. Bies [79]. The reporter construct p6MBSluc containing six Myb-binding sites upstream of luciferase cDNA was kindly provided by T. Nakano [80]. CMV-βgal was described elsewhere [81]. Reporter constructs with cathepsin D promoter sequences (2.85 and 0.82 kb) in pGL3 were kindly provided by J. Chirgwin [82]. Reporter constructs containing the MMP1 promoter sequences (4.3-1 G, 1.606, and 0.624 kb) in pGL3 were kindly provided by C. E. Brinckerhoff [83] and A. Galloway [84]. The MMP9 promoter sequence (-1362/+19) was obtained from the genomic DNA of human blood cells by PCR using the forward primer 5'-GCGCGCAGATCTTATAGACCCTGCCCGATGCCGG-3' and the reverse primer 5'-GCGCGCAAGCTTTGGTGAGGGCAGAGGTGTCTGAC-3'. The PCR product was cut with *Bgl*II/*Hind*III and ligated into pGL2-basic (Promega, Madison, WI).

### Cells and transfection

Human MDA-MB-231 and mouse mammary carcinoma 4T1 cells obtained from American Type Culture Collection were cultured in RPMI 1640 (Sigma, St. Louis, MO) supplemented with 10% FCS, 2 mM L-glutamine 100 U/ml penicillin and 100 μg/ml streptomycin. Medium for 4T1 cells was supplemented with 4500 mg/l glucose and 1 mM sodium pyruvate. MDA-MB-231 cells were transfected with the pcDNA3-hcMYB plasmid and the *myb*-less control plasmid. 4T1 cells were transfected with the pcDNA3mcMYB plasmid and the *myb*-less control plasmid. Transfections were performed using Lipofectamine 2000 (Invitrogen, Carlsbad, CA) according to the manufacturer's instructions. Transfectants were selected with 800 μg/ml G418 (MDA-MB-231 cells) and 300 μg/ml G418 (4T1 cells) and cloned by limiting dilution.

### Transmigration/invasion assay

Cell migration and invasion were analyzed using Cultrex cell invasion assay (Trevigen, Gaithersberg, MD) according to the manufacturer's instructions. Briefly, for the invasion assay, the membrane in the upper chamber of 96-well plate was coated with 0.5 × basement membrane matrix/Matrigel. For the transmigration assay, the membrane was left uncoated. MDA-MB-231 cells were starved in the serum-free medium for 8 hours prior assay, then seeded at a density 5 × 10^4^/well. After 16 h, the cells on the lower surface were dissociated and stained with the Calcein-AM. The amount of emitted fluorescence was determined by Synergy HT microplate reader (Bio-tek, Winooski, VT) to quantify the relative cell migration/invasion.

### Kinetics of cell migration/invasion

To monitor cell migration/invasion in real time, we used the xCELLigence Real-Time Cell Analyzer (RTCA) DP Instrument equipped with a CIM-plate 16 (Roche, Indianapolis, IN). The CIM-plate 16 is a 16-well system in which each well is composed of upper and lower chambers separated by an 8-μm microporous membrane. Migration/invasion was measured as the relative impedance change (cell index) across microelectronic sensors integrated into the bottom side of the membrane. For the cell invasion experiments, the membrane was coated with either Matrigel (BD Biosciences, San José, CA) or collagen I (Sigma, St. Louis, MO). Matrigel was diluted 1:40 in serum-free medium and allowed to polymerize at 37°C for 4 h. The collagen solution was neutralized with NaOH and allowed to form a 3D gel at 37°C for 1 h. For the cell migration experiments, the membrane was left uncoated. FCS (10%) was used as a chemoattractant. Cells were starved in a serum-free media for 8 h. Cells (7.5 × 10^4^) were added in duplicates to the upper chambers. Migration/invasion was monitored every 10 or 15 min for several hours. For quantification, the cell index at indicated time points was averaged from at least three independent measurements.

### RNA isolation and quantitative RT-PCR

Total RNA was isolated from MDA-MB-231 cells using an RNeasy mini kit (Qiagen, Valencia, CA) according to the manufacturer's instructions. RNA (0.5 μg) was used for first-strand cDNA synthesis with the QuantiTect Reverse Transcription kit (Qiagen, Valencia, CA). Human *MMP2*, *3, 7, 10, 11, 13 *mRNA were quantified by qRT-PCR with specific primers and RT^2 ^SYBR Green/ROX PCR Master Mix (SABiosciences, Frederick, MD). Human c-*myb*, *cathepsin D*, *MMP9*, and *MMP1 *mRNAs were quantified by qRT-PCR using specific TaqMan probes (Applied Biosystems, Foster City, CA). *GAPDH *was used as an internal control. All PCR reactions were performed in triplicate for each sample and were repeated at least three times. The qRT-PCR data were analyzed by the ΔΔCt method.

### Immunoblotting

Cell lysates were subjected to SDS-PAGE and immunoblotted as previously described [85]. The conditioned media were concentrated using Amicon Ultra Centrifugal Filters with the Ultracel-10 membrane (Millipore, Billerica, MA). Protein concentrations were determined using the DC protein assay (Bio-Rad, Hercules, CA). Equal amounts of total protein (30 μg) were loaded. Blots were probed with anti-Myb (05-175, Millipore, Billerica, MA), anti-cathepsin D (610801, BD Biosciences, San José, CA), anti-MMP1 (1976-S, EpiSelect MMP sampler kit, Epitomics, Burlingame, CA), and anti-MMP9 (G657, Cell Signaling Technology, Beverly, MA) antibodies. To control for sample loading, the blots were probed with a β-actin-specific antibody (A5060, Sigma, St. Louis, MO). Blots were developed by standard ECL procedure using Immobilon Western Chemiluminiscent HRP Substrate (Millipore, Billerica, MA).

### Cathepsin D assay

The cells (9 × 10^5^) were collected in 80 μl of sterile-filtered water and subjected to three freeze-thaw cycles. After centrifugation at 25 000 × *g *for 5 min, 20 μg of cell extracts were subjected to the Cathepsin D Assay (Sigma, St. Louis, MO) according to the manufacturer's instructions. Pepstatin A was added to determine the cathepsin D-specific fluorescence signals. The plates were incubated at 37°C for 30 min, and readings were performed in 2-min intervals using a Synergy HT microplate reader (Bio-tek, Winooski, VT).

### Transactivation assay

To determine transactivation by c-Myb, MDA-MB-231 wt, vector, and MYBup cells were transiently cotransfected with p6MBSluc/pGL3-CD/pGL2-MMP9/pGL3-MMP1 and CMV-βgal plasmids using Lipofectamine 2000 (Invitrogen, Carlsbad, CA) according to the manufacturer's instructions. Cells were cultured for 24 h and processed for luciferase and β-galactosidase assays as described elsewhere [86]. The luciferase activity of each sample was expressed in relative light units and normalized for transfection efficiency according to the β-galactosidase activity.

### *Cathepsin D*/c-*myb *knockdown using siRNA

The *cathepsin D *siRNA oligos (5'-GGAUCCCGCUGCACAAGUUTT-3') were purchased from Ambion (Austin, TX). The c-*myb *siRNA oligos (5'-UAUAGUGUCUCUGAAUGGCUGCGGC-3') were purchased from Invitrogen. ON-TARGETplus nontargeting pool siRNA (Dharmacon, Lafayette, CO) was used as a negative control. Transfection with siRNA was performed using xtremeGENE siRNA transfection reagent according to the manufacturer's recommendations (Roche, Indianapolis, IN). siRNA (30 nM, final concentration) was added to the plates for 3 h. After 72 h, the cells were processed for a cell migration assay as described above. At the same time, the cells were harvested, and cathepsin D/c-Myb expression was determined by immunoblotting.

### Metastasis assay

To evaluate the formation of spontaneous metastases, c-*myb-*overexpressing and control 4T1 tumor cells (1 × 10^5 ^in a 20-μl Matrigel:PBS solution, 1:1) were injected into the fourth mammary fat pad of female BALB/c mice aged 6-8 weeks. The use of these animals followed the European Community Guidelines as accepted principles for the use of experimental animals. The experiments were performed with the approval of the Institutional Ethical Committee. Tumor growth was monitored at least twice a week by measuring the tumor length (l) and width (w) with caliper, and tumor volume was calculated using the equation l × w^2^/2. The mice were euthanized when the mean tumor diameter was approximately 1.2 cm [87]. Tumors were excised and weighed. Lungs were fixed in Bouin's solution, and liver and bones were fixed in 10% buffered formalin. Surface metastatic nodules in lungs were determined by dissecting microscopy. Lungs, liver and bone tissues were processed for paraffin embedding, sectioned, and stained with hematoxylin and eosin (H&E). Bones were decalcified overnight before embedding. The number of pulmonary metastatic lesions was determined by histological examination. Every 10th consecutive section was examined for the presence of metastases. The extent of liver and bone metastases was evaluated semi-quantitatively by light microscopy.

### Statistics

Values were expressed as means ± standard deviations. To determine statistical significance, the values were compared by unpaired two-tailed *t*-test. Differences were considered to be significant at p < 0.05.

## Abbreviations

BM: Basement membrane; EMT: Epithelial-to-mesenchymal transition; ECM: Extracellular matrix; MMP: Matrix metalloproteinase; RTCA: Real-time cell analyzer.

## Competing interests

The authors declare that they have no competing interests.

## Authors' contributions

LK and PB carried out experiments *in vitro *and analyzed results as well as took part in writing the manuscript. LP participated in performing transactivation assays. MM, LK, KS and ZP participated in *in vivo *experiments. MH provided histological analysis. JS supervised the experimental work, participated in data analysis and interpretation of results and revised the manuscript. All authors read and approved the manuscript.

## Supplementary Material

Additional file 1 **Figure S1 Kinetics of c-Myb-induced migration and Matrigel invasion of MDA-MB-231MYBup cells**. Cell migration/invasion was analyzed in real time by the xCELLigence RTCA as described in Figure 3. The panels show the average cell indexes at indicated time points from seven (migration) and five (invasion) independent experiments. Error bars indicate standard deviations. Asterisks indicate significant (p < 0.05) differences in the migration/Matrigel invasion of the *myb*-less vector-transfected cells and MYBup cells (M2, M5) as determined by the *t*-test. Click here for file

Additional file 2 **Figure S2 siRNA-mediated c-*myb *silencing reduces migration and Matrigel invasion activities of MDA-MB-231MYBup cells**. MDA-MB-231MYBup cells were transfected with c-*myb *(MYB) or control (ctrl) siRNAs as described in the Material and Methods. The level of c-Myb protein in these cells was determined by immunoblotting. A representative western blot is presented (**A**). Migration and invasion activities of the same cells were determined by the xCELLigence RTCA. The chart shows the representative outcomes of the kinetics analysis of cell migration (**B**). The average cell indexes at the 6-h (migration, left) and 12-h (invasion, right) time points, respectively, from four independent measurements are shown (**C**). Error bars indicate standard deviations. Asterisks indicate significant (p < 0.05) differences in the migration/invasion rates of the cells transfected with c-*myb *siRNA and control siRNA as determined by the *t*-test. Click here for file

Additional file 3 **Figure S3 c-Myb-induced Matrigel invasion is sensitive to the MMP inhibitor GM6001**. MDA-MB-231MYBup cells (M2, M5) were starved in the serum-free medium for 8 h and processed for cell invasion assay as described in the Material and Methods. The membranes were coated with the diluted Matrigel. The broad-spectrum MMP inhibitor GM6001 (Sigma) at final concentration of 20 μM or DMSO were added to the bottom and upper chambers of indicated wells of CIM-plate 16. The bottom chambers were filled with complete medium containing 10% FCS as a chemoattractant. (**A**) The chart shows the representative outcomes of kinetics analysis of Matrigel invasion. (**B**) The columns show the average cell indexes in 12 h time point from three independent measurements. Error bars indicate standard deviations. Asterisks indicate significant (p < 0.05) differences in Matrigel invasion of the GM6001-treated MDA-MB-231MYBup cells and DMSO-treated MDA-MB-231MYBup controls as determined by the *t*-test. Click here for file

Additional file 4 **Figure S4 Histological examination of lungs, liver and bones of BALB/c mice orthotopically injected with c-*myb *overexpressing (MYBup) and control 4T1 cells**. Lungs were fixed in Bouin's solution. Liver and bones were harvested and fixed in 10% buffered formalin. Tissues were processed for paraffin embedding, sectioned, and stained with hematoxylin and eosin. Bones were decalcified overnight before embedding. (**A**) The mean number of pulmonary metastatic lesions as determined by histological examination. Every 10th consecutive section was examined for the presence of metastases (mts). (**B**) Semi-quantitative evaluation of liver metastasis: + rare microscopic neoplastic lesions, ++ numerous microscopic lesion, +++ numerous extensive neoplastic lesions. (**C**) Semi-quantitative evaluation of skeletal metastasis: + epithelial tumor cell infiltration and bone destruction, ++ tumor cell infiltration and bone destruction with the invasion of soft tissues. Click here for file

Additional file 5 **Figure S5 c-Myb upregulates Slug in MDA-MB-231MYBup cells**. The nontransfected (wt), the *myb*-less vector-transfected (vector) and MYBup (M2, M5) cells were harvested. Protein extracts were resolved by SDS-PAGE and analyzed by immunoblotting with anti-Slug (9585, Cell Signaling), anti-vimentin (13.2, Sigma) and anti-N-cadherin (610920, BD Biosciences) antibodies. To control for sample loading, the blots were probed with the β-actin-specific antibody. Click here for file

Additional file 6 **Figure S6 c-Myb induces the *cathepsin D *transcription independently on Slug**. MDA-MB-231 cells were transiently cotransfected with either c-*myb *(MYBup)*, Slug *cDNA (SLUGup) or the empty vector (vector) and the reporter plasmids (the 2.85 kb- or 0.82 kb pGL3-CD containing segments of *cathepsin D *promoter of indicated lengths upstream of the *luciferase *gene, and CMV-βgal) and harvested 48 h later. (**A**) The amounts of c-Myb and Slug proteins were determined by immunoblotting. (**B**) The luciferase activity of each sample was expressed in relative light units and normalized for transfection efficiency according to the β-galactosidase activity. The columns show the average relative luciferase activity from three independent measurements. Error bars indicate the standard deviations. Asterisks indicate significant (p < 0.05) differences in the luciferase activity in the empty vector-transfected cells and cells transfected with c-*myb *cDNA as determined by the *t*-test. Click here for file

Additional file 7 **Figure S7 TESS analysis of 2.5 kb promoter region of human cathepsin D gene**. Lines indicate putative Myb-binding sites identified with the Transcription Element Search Software (TESS; http://www.cbil.upenn.edu/cgi-bin/tess/). Click here for file

Additional file 8 **Figure S8a c-Myb down-regulates interstitial collagenase Mmp1a in 4T1 cells**. The control (wt, vector) and the MYBup (M-M5, M-M8) cell protein extracts were analyzed by immunoblotting. To control for sample loading, the blots were probed with a β-actin-specific antibody. The secretion of Mmp1a was determined in the cell-conditioned medium. The cells were cultured in a serum-free medium for 8 h, and the medium was harvested and concentrated using the Amicon Ultra centrifugal filter units. Equal amounts of total proteins were loaded. **Figure S8b **Expression of c-*myb *and *mmp1a *mRNAs in 4T1 MYBup and control tumors. Total RNA was isolated from mouse mammary tumors. The relative amounts of c-*myb *and *mmp1a *mRNAs in the MYBup and control tumors were determined by qRT-PCR. *GAPDH *was used as an internal control. Asterisks indicate significant (p < 0.05) differences in the relative amounts of c-*myb *and *mmp1a *mRNAs in the control and MYBup tumors as determined by the *t*-test. Click here for file
