# Supplementary material for: c-Myb regulates matrix metalloproteinases 1/9, and cathepsin D: implications for matrix-dependent breast cancer cell invasion and metastasis
Source: Mol Cancer. 2012 Mar 23;11:15. doi: 10.1186/1476-4598-11-15 (PMC3325857; doi:10.1186/1476-4598-11-15)
Supplement: Additional file 1 — Figure S1 Kinetics of c-Myb-induced migration and Matrigel invasion of MDA-MB-231MYBup cells. Cell migration/invasion was analyzed in real time by the xCELLigence RTCA as described in Figure 3. The panels show the average cell indexes at indicated time points from seven (migration) and five (invasion) independent experiments. Error bars indicate standard deviations. Asterisks indicate significant (p < 0.05) differences in the migration/Matrigel invasion of the myb-less vector-transfected cells and MYBup cells (M2, M5) as determined by the t-test. [file 1476-4598-11-15-S1.PDF]

Additional file 1:

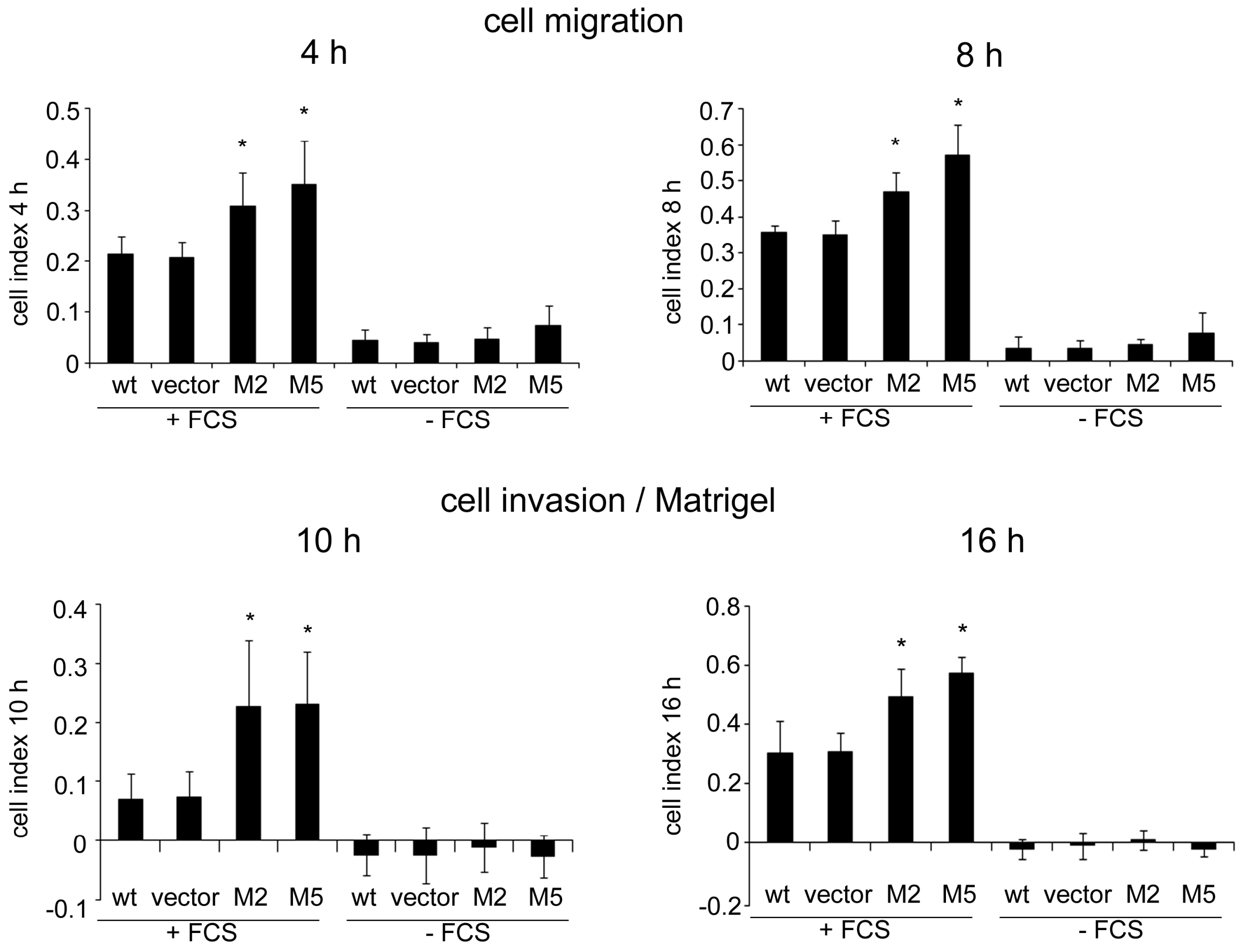

Figure S1. Kinetics of c-Myb-induced migration and Matrigel invasion of MDA-MB-231MYBup cells. Cell migration/invasion in real time was analyzed by the xCELLigence RTCA as described in Figure 3. The panels show the average cell indices at certain time points from seven (migration) and five (invasion) independent measurements: (**A**) 4 h and (**B**) 8 h for cell migration; (**C**) 10 h and (**D**) 16 h for cell invasion. Error bars indicate standard deviations. Asterisks indicate significant ($p<0.05$) differences in the migration/Matrigel invasion of the *myb*-less vector-transfected cells and MYBup cells (M2, M5) as determined by the *t*-test.
