# Supplementary material for: c-Myb regulates matrix metalloproteinases 1/9, and cathepsin D: implications for matrix-dependent breast cancer cell invasion and metastasis
Source: Mol Cancer. 2012 Mar 23;11:15. doi: 10.1186/1476-4598-11-15 (PMC3325857; doi:10.1186/1476-4598-11-15)
Supplement: Additional file 2 — Figure S2 siRNA-mediated c-myb silencing reduces migration and Matrigel invasion activities of MDA-MB-231MYBup cells. MDA-MB-231MYBup cells were transfected with c-myb (MYB) or control (ctrl) siRNAs as described in the Material and Methods. The level of c-Myb protein in these cells was determined by immunoblotting. A representative western blot is presented (A). Migration and invasion activities of the same cells were determined by the xCELLigence RTCA. The chart shows the representative outcomes of the kinetics analysis of cell migration (B). The average cell indexes at the 6-h (migration, left) and 12-h (invasion, right) time points, respectively, from four independent measurements are shown (C). Error bars indicate standard deviations. Asterisks indicate significant (p < 0.05) differences in the migration/invasion rates of the cells transfected with c-myb siRNA and control siRNA as determined by the t-test. [file 1476-4598-11-15-S2.PDF]

**Additional file 2:**

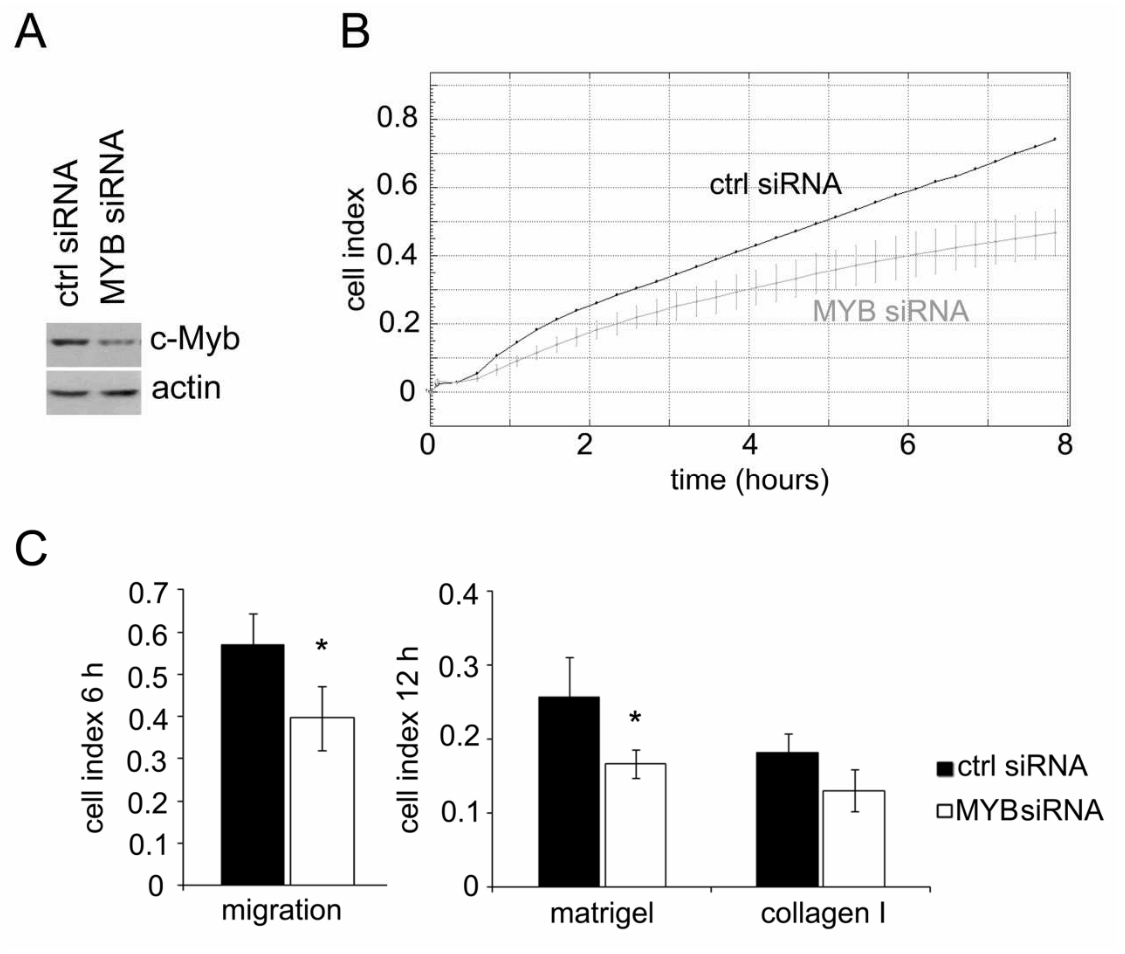


**Figure S2. SiRNA-mediated c-*myb* silencing reduces migration and Matrigel invasion activities of MDA-MB-231MYBup cells.** MDA-MB-231MYBup cells were transfected with c-*myb* (MYB) or control (ctrl) siRNAs as described in the Material and Methods. The level of c-Myb protein in these cells was determined by immunoblotting. A representative western blot is presented (**A**). Migration and invasion activities of the same cells were determined by the xCELLigence RTCA. The chart shows the representative outcomes of the kinetics analysis of cell migration (**B**). The average cell indexes at the 6-h (migration, left) and 12-h (invasion, right) time points, respectively, from four independent measurements are shown (**C**). Error bars indicate standard deviations. Asterisks indicate significant ($p<0.05$) differences in the migration/invasion rates of the cells transfected with c-*myb* siRNA and control siRNA as determined by the *t*-test.
