# Supplementary material for: c-Myb regulates matrix metalloproteinases 1/9, and cathepsin D: implications for matrix-dependent breast cancer cell invasion and metastasis
Source: Mol Cancer. 2012 Mar 23;11:15. doi: 10.1186/1476-4598-11-15 (PMC3325857; doi:10.1186/1476-4598-11-15)
Supplement: Additional file 3 — Figure S3 c-Myb-induced Matrigel invasion is sensitive to the MMP inhibitor GM6001. MDA-MB-231MYBup cells (M2, M5) were starved in the serum-free medium for 8 h and processed for cell invasion assay as described in the Material and Methods. The membranes were coated with the diluted Matrigel. The broad-spectrum MMP inhibitor GM6001 (Sigma) at final concentration of 20 μM or DMSO were added to the bottom and upper chambers of indicated wells of CIM-plate 16. The bottom chambers were filled with complete medium containing 10% FCS as a chemoattractant. (A) The chart shows the representative outcomes of kinetics analysis of Matrigel invasion. (B) The columns show the average cell indexes in 12 h time point from three independent measurements. Error bars indicate standard deviations. Asterisks indicate significant (p < 0.05) differences in Matrigel invasion of the GM6001-treated MDA-MB-231MYBup cells and DMSO-treated MDA-MB-231MYBup controls as determined by the t-test. [file 1476-4598-11-15-S3.PDF]

Additional file 3:

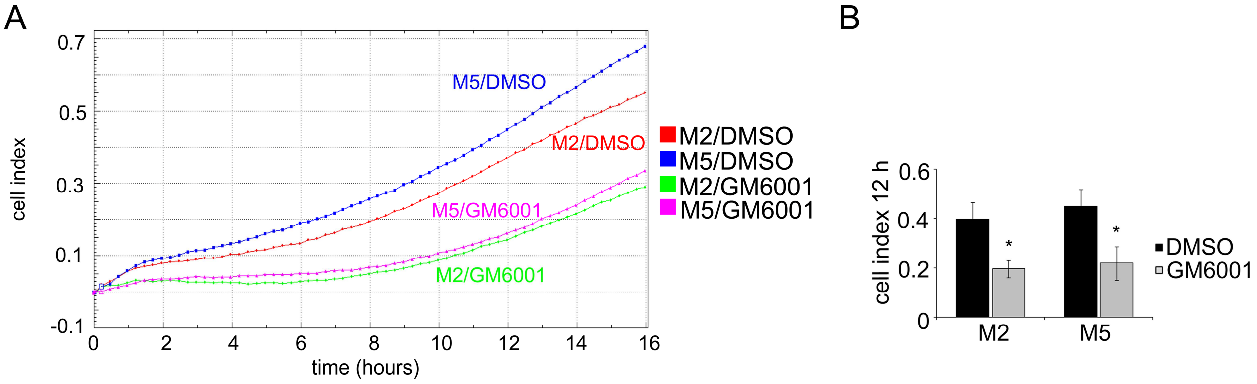

Figure S3. **c-Myb-induced Matrigel invasion is sensitive to the MMP inhibitor GM6001.** MDA-MB-231MYBup cells (M2, M5) were starved in the serum-free medium for 8 h and processed for cell invasion assay as described in the Material and Methods. The membranes were coated with the diluted Matrigel. The broad-spectrum MMP inhibitor GM6001 (Sigma) at final concentration 20 µM or DMSO was added to the bottom and upper chambers of indicated wells of CIM-plate 16. The bottom chambers were filled with complete medium containing 10% FCS as a chemoattractant. (**A**) The chart shows the representative outcomes of kinetics analysis of Matrigel invasion. (**B**) The columns show the average cell indexes in certain time points (12 h) from three independent measurements. Error bars indicate standard deviations. Asterisks indicate significant ($p<0.05$) differences in Matrigel invasion of the GM6001-treated MDA-MB-231MYBup cells and DMSO-treated MDA-MB-231MYBup controls as determined by the *t*-test.
