# Supplementary material for: c-Myb regulates matrix metalloproteinases 1/9, and cathepsin D: implications for matrix-dependent breast cancer cell invasion and metastasis
Source: Mol Cancer. 2012 Mar 23;11:15. doi: 10.1186/1476-4598-11-15 (PMC3325857; doi:10.1186/1476-4598-11-15)
Supplement: Additional file 4 — Figure S4 Histological examination of lungs, liver and bones of BALB/c mice orthotopically injected with c-myb overexpressing (MYBup) and control 4T1 cells. Lungs were fixed in Bouin's solution. Liver and bones were harvested and fixed in 10% buffered formalin. Tissues were processed for paraffin embedding, sectioned, and stained with hematoxylin and eosin. Bones were decalcified overnight before embedding. (A) The mean number of pulmonary metastatic lesions as determined by histological examination. Every 10th consecutive section was examined for the presence of metastases (mts). (B) Semi-quantitative evaluation of liver metastasis: + rare microscopic neoplastic lesions, ++ numerous microscopic lesion, +++ numerous extensive neoplastic lesions. (C) Semi-quantitative evaluation of skeletal metastasis: + epithelial tumor cell infiltration and bone destruction, ++ tumor cell infiltration and bone destruction with the invasion of soft tissues. [file 1476-4598-11-15-S4.PDF]

Additional file 4:

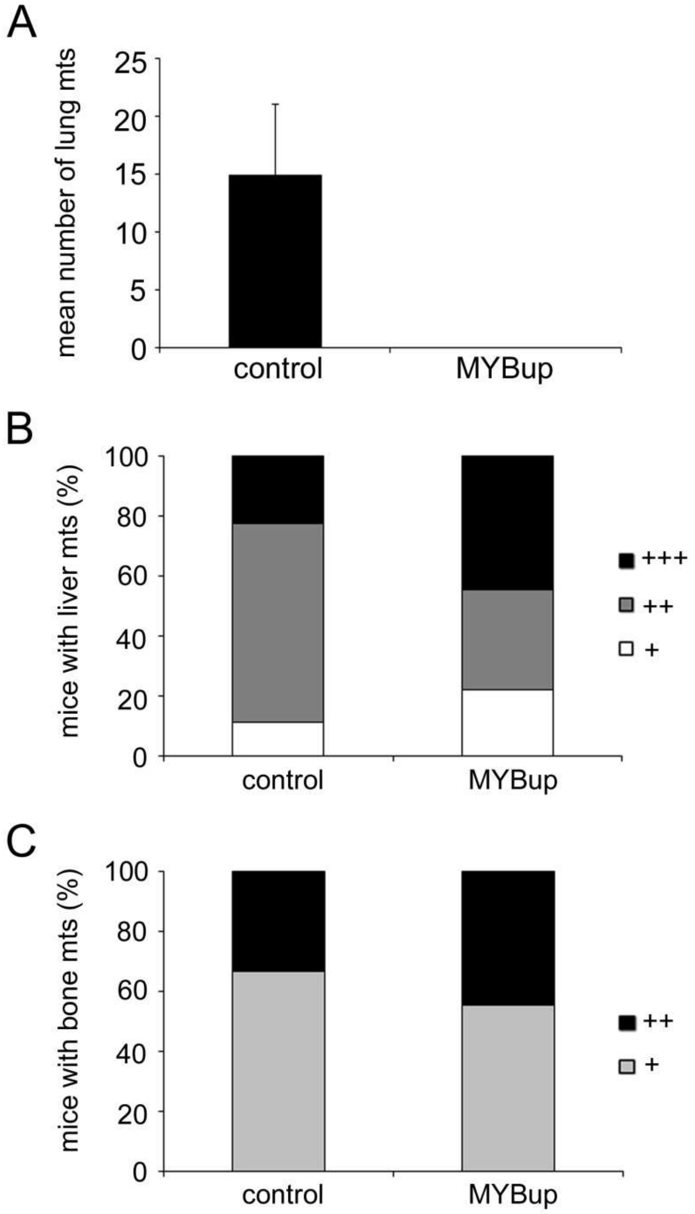

Figure S4. Histological examination of lungs, liver and bones of BALB/c mice orthotopically injected with c-*myb* overexpressing (MYBup) or control 4T1 cells. Lungs were fixed in Bouin's solution. Liver and bones were harvested and fixed in 10% buffered formalin. Tissues were processed for paraffin embedding, sectioned, and stained with hematoxylin and eosin (H&E). Bones were decalcified overnight before embedding. (**A**) The mean number of pulmonary metastatic lesions as determined by histological examination. Every 10th consecutive section was examined for the presence of metastases (mts). (**B**) Semi-quantitative evaluation of liver metastasis: + rare microscopic neoplastic lesions, ++ numerous microscopic lesion, +++ numerous extensive neoplastic lesions. (**C**) Semi-quantitative evaluation of skeletal metastasis: + epithelial tumor cell infiltration and bone destruction, ++ tumor cell infiltration and bone destruction with the invasion of soft tissues.
