# Supplementary material for: c-Myb regulates matrix metalloproteinases 1/9, and cathepsin D: implications for matrix-dependent breast cancer cell invasion and metastasis
Source: Mol Cancer. 2012 Mar 23;11:15. doi: 10.1186/1476-4598-11-15 (PMC3325857; doi:10.1186/1476-4598-11-15)
Supplement: Additional file 5 — Figure S5 c-Myb upregulates Slug in MDA-MB-231MYBup cells. The nontransfected (wt), the myb-less vector-transfected (vector) and MYBup (M2, M5) cells were harvested. Protein extracts were resolved by SDS-PAGE and analyzed by immunoblotting with anti-Slug (9585, Cell Signaling), anti-vimentin (13.2, Sigma) and anti-N-cadherin (610920, BD Biosciences) antibodies. To control for sample loading, the blots were probed with the β-actin-specific antibody. [file 1476-4598-11-15-S5.PDF]

Additional file 5:

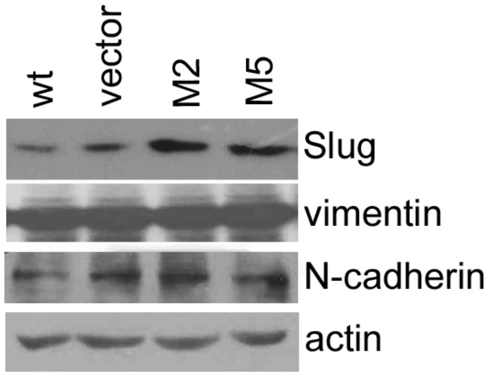

Figure S5. c-Myb upregulates Slug in MDA-MB-231MYBup cells. The nontransfected (wt), the *myb*-less vector-transfected (vector) and MYBup (M2, M5) cells were harvested. Protein extracts were resolved by SDS-PAGE and analyzed by immunoblotting with anti-Slug (9585, Cell Signaling), anti-vimentin (13.2, Sigma) and anti-N-cadherin (610920, BD Biosciences) antibodies. To control for sample loading, the blots were probed with the β-actin-specific antibody.
