# Supplementary material for: c-Myb regulates matrix metalloproteinases 1/9, and cathepsin D: implications for matrix-dependent breast cancer cell invasion and metastasis
Source: Mol Cancer. 2012 Mar 23;11:15. doi: 10.1186/1476-4598-11-15 (PMC3325857; doi:10.1186/1476-4598-11-15)
Supplement: Additional file 6 — Figure S6 c-Myb induces the cathepsin D transcription independently on Slug. MDA-MB-231 cells were transiently cotransfected with either c-myb (MYBup), Slug cDNA (SLUGup) or the empty vector (vector) and the reporter plasmids (the 2.85 kb- or 0.82 kb pGL3-CD containing segments of cathepsin D promoter of indicated lengths upstream of the luciferase gene, and CMV-βgal) and harvested 48 h later. (A) The amounts of c-Myb and Slug proteins were determined by immunoblotting. (B) The luciferase activity of each sample was expressed in relative light units and normalized for transfection efficiency according to the β-galactosidase activity. The columns show the average relative luciferase activity from three independent measurements. Error bars indicate the standard deviations. Asterisks indicate significant (p < 0.05) differences in the luciferase activity in the empty vector-transfected cells and cells transfected with c-myb cDNA as determined by the t-test. [file 1476-4598-11-15-S6.PDF]

Additional file 6:

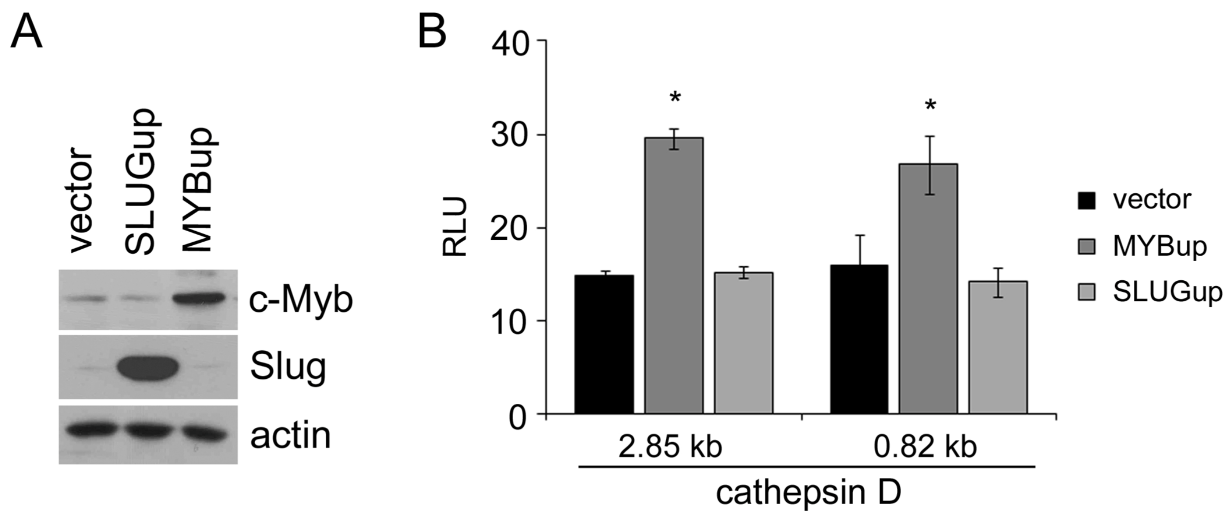

Figure S6. c-Myb induces the *cathepsin D* transcription independently on Slug. MDA-MB-231 cells were transiently cotransfected with either c-*myb* (MYBup), *Slug* cDNA (SL UGup) or the empty vector (vector) and the reporter plasmids (the 2.85 kb- or 0.82 kb pGL3-CD containing segments of *cathepsin D* promoter of indicated lengths upstream of the *luciferase* gene, and CMV-βgal) and harvested 48 h later. (**A**) The amounts of c-Myb and Slug proteins were determined by immunoblotting. (**B**) The luciferase activity of each sample was expressed in relative light units and normalized for transfection efficiency according to the β-galactosidase activity. The columns show the average relative luciferase activity from three independent measurements. Error bars indicate the standard deviations. Asterisks indicate significant ($p<0.05$) differences in the luciferase activity in the empty vector-transfected cells and cells transfected with c-*myb* cDNA as determined by the *t*-test.
