# Supplementary material for: c-Myb regulates matrix metalloproteinases 1/9, and cathepsin D: implications for matrix-dependent breast cancer cell invasion and metastasis
Source: Mol Cancer. 2012 Mar 23;11:15. doi: 10.1186/1476-4598-11-15 (PMC3325857; doi:10.1186/1476-4598-11-15)
Supplement: Additional file 7 — Figure S7 TESS analysis of 2.5 kb promoter region of human cathepsin D gene. Lines indicate putative Myb-binding sites identified with the Transcription Element Search Software (TESS; http://www.cbil.upenn.edu/cgi-bin/tess/). [file 1476-4598-11-15-S7.PDF]

Additional file 7:

Cathepsin D promoter sequence with Myb-binding sites

gggaccttcctgcggaggtcgctgttgagcacatcccctggctccatttcctgtgtgacttggacctgccttaaa
tgatttgtcccaatatccctttcctacttacagcgaccgcacctcccgcctggctgtgtgctccagagcatctgg
ggaatgaccgggccctcccctccaacaggcctaattcagtcccccatgccaggcaggaccagggcacacgagaat
ggtgaagtcctgggg**caactg**tcaccatggcctctgcaagctcagtgtcgggagacccagtttctgacctggata
cactgctcacctttgaggtcggggcctagagggacggtgccacgttggagacaggcctccagggacagtgccacg
ttgaagacggggttccagggacggtgccac**gtta**gagatggggttccagggacagtgccacgttgaagacggggt
tccagggacggtgccacgttggagatggggttccagggatggtgccacattggagatgggcaagtctgggctaag
ggaggaggcccagggtggccagggcagggcccagtgaggtgctgctctgaggaggtggagagtgggcgcttgggc
aggggacacttggagagtaaggatgaggtccatctgggtaccctgggtatagggagtgttgttgaaaaaaagcca
aacactataaaatatttgaagatatttattctgagcaaatgtgaggactatgacctgtgaaaccacctcagcagg
tcctgagaatatgtgagcaaggtggctggg**taac**agcttggctttatacttttttttaaaattttcaggtctttt
ctgtagagacagggtctcaccatgttgcccaggctgatctcaagctcttcagctcaagcaatgctcccaccttgg
cctccgagtatgctgggattataggcaacagtcaccatgcccggcctggttttacggattttacagagacagatg
ttacaggccaaaaccataaatcaacatatgtaagatatacattggttcagcctagaaacagggttggcaggggca
gcttccaggtcataggggattcaaagatttcctgattggcaagtggttgaaaga**gtta**tgttttgcctaaaga**gt**
taaagtgggattactcacgcctgtaatcccagcactttggaaggctgaggcaggcagatcacctgaggtcaggag
ttcgagaccagcctggccaacatggtgaaacaccatctctactaaaaatacaaaacatatatacacatatacatg
tatatatatatacacatatacatgtatatatatacacatatacatgtatatatatacacatatacatgtatatat
atacatatatacatgtatatatatacacatatacatgtatatatatacacatatatacacatatacatgtatata
tacacatatatacacatatacatatatgtatatacatatatagtgtatatatatatacgtatatatatgtgtata
tatacactatatatgtgtgtgtgtgtatatatatatatacatacgtgtgtgtatatatacatattagccaggcgt
ggtagcacgcgcctgtaatcccagctacttaggaggctgaggcaggagaatcgcttgaacccaggaggtagaggt
tgcagtgagccgagatcatgccactgcactccagcctgggccacagagcgagattctgtctcaaaaaaaaaaaaa
aaaaaaaagagttgacgtgagtggacaaaaggtgcccgacctcctaagggaaggagcttctctagaaaacgcgaa
tttcgcccccctcaagagacagctgtgcagtgccatatcaaaacatgtgaaaggaatgtattttagggtggaatac
tttgcctgccttcgggcctgctgtctgccacgtgaggctgtgcagtgtgaggctggaatttgggatctggaggct
agagccatcggtgaggcctgagtctctaagcacagcgcccagaggagagggcggacgggtccgaccccctttgcg
gcagggcctgagctggttttccaggtttctctggaagccctgtagaggagcggagggtccattcggtgggctggg
gactttgaatt**taac**cttggtttgcaagaggcttccagagaggatgtctgggagcgtctcggagggggacgaggg
ggcgccgggaggagcaggtgcaggagcccacggcgcacgccccgcgcaggcctggacgcggggacggccgcggcg
gccgggacaggggtcaccccgcggggccctccagggtgggccgccccacgaccccgggccaggccgaaacgggaa
tcctccagaccccagaagctgggccgggctgaccccgcgggcgcgagcggcgggaactgtaggcgcggcaggcgc
accaccaccccccccccgcccgggcgctgtgcgcgtgcgccgaggttgccccgcccaggccaggccccgtccgcc
ccgccccgcgcacgccggccgcgcccacgtgaccggtccgggtgcaaacacgcgggtcagctgatccggcc**caac**
tgcggcgtcatcccggctataagcgcacggcctcggcgaccctctccgacccggccgccgccgccatg

Figure S7. TESS analysis of 2.5kb promoter region of human cathepsin D gene. Lines indicate putative Myb-binding sites identified with the Transcription Element Search Software (TESS; http://www.cbil.upenn.edu/cgi-bin/tess/).
