# Supplementary material for: c-Myb regulates matrix metalloproteinases 1/9, and cathepsin D: implications for matrix-dependent breast cancer cell invasion and metastasis
Source: Mol Cancer. 2012 Mar 23;11:15. doi: 10.1186/1476-4598-11-15 (PMC3325857; doi:10.1186/1476-4598-11-15)
Supplement: Additional file 8 — Figure S8a c-Myb down-regulates interstitial collagenase Mmp1a in 4T1 cells. The control (wt, vector) and the MYBup (M-M5, M-M8) cell protein extracts were analyzed by immunoblotting. To control for sample loading, the blots were probed with a β-actin-specific antibody. The secretion of Mmp1a was determined in the cell-conditioned medium. The cells were cultured in a serum-free medium for 8 h, and the medium was harvested and concentrated using the Amicon Ultra centrifugal filter units. Equal amounts of total proteins were loaded. Figure S8b Expression of c-myb and mmp1a mRNAs in 4T1 MYBup and control tumors. Total RNA was isolated from mouse mammary tumors. The relative amounts of c-myb and mmp1a mRNAs in the MYBup and control tumors were determined by qRT-PCR. GAPDH was used as an internal control. Asterisks indicate significant (p < 0.05) differences in the relative amounts of c-myb and mmp1a mRNAs in the control and MYBup tumors as determined by the t-test. [file 1476-4598-11-15-S8.PDF]

Additional file 8:

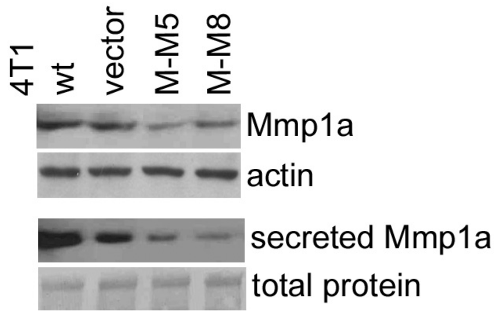

Figure S8a. **c-Myb down-regulates interstitial collagenase Mmp1a in 4T1 cells**. The control (wt, vector) and the MYBup (M-M5, M-M8) cell protein extracts were analyzed by immunoblotting. To control for sample loading, the blots were probed with a β-actin-specific antibody. The secretion of Mmp1a was determined in the cell-conditioned medium. The cells were cultured in a serum-free medium for 8 h, and the medium was harvested and concentrated using the Amicon Ultra centrifugal filter units. Equal amounts of total proteins were loaded.

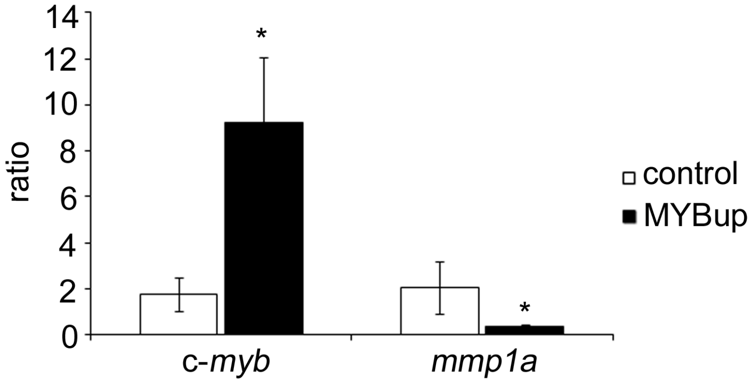

Figure S8b. **Expression of c-*myb* and *mmp1a* mRNAs in 4T1 MYBup and control tumors.** Total RNA was isolated from mouse mammary tumors. The relative amounts of c-*myb* and *mmp1a* mRNAs in the MYBup and control tumors were determined by qRT-PCR. *GAPDH* was used as an internal control. Asterisks indicate significant ($p<0.05$) differences in the relative amounts of c-*myb* and *mmp1a* mRNAs in the control and MYBup tumors as determined by the *t*-test.
